# Routine Monitoring of Instrument Stability in a Milk Testing Laboratory With ASCA: A Pilot Study

**DOI:** 10.3389/fchem.2021.733331

**Published:** 2021-10-07

**Authors:** Michel K. Nieuwoudt, Cannon Giglio, Federico Marini, Gavin Scott, Stephen E. Holroyd

**Affiliations:** ^1^ The Photon Factory, The University of Auckland, Auckland, New Zealand; ^2^ School of Chemical Sciences, The University of Auckland, Auckland, New Zealand; ^3^ The MacDiarmid Institute for Advanced Materials and Nanotechnology, Wellington, New Zealand; ^4^ The Dodd-Walls Centre for Photonic and Quantum Technologies, Dunedin, New Zealand; ^5^ Fonterra On-farm R and D, Hamilton, New Zealand; ^6^ Dipartimento di Chimica, Universita di Roma “La Sapienza”, Rome, Italy; ^7^ Fonterra Research and Development, Palmerston North, New Zealand

**Keywords:** ASCA, quality control, milk testing, instrument stability, standardization, FTIR spectroscopy, analysis of variance with simultaneous component analysis

## Abstract

Mid-infrared spectroscopy has been developed as a reliable and rapid tool for routine analysis of fat, protein, lactose and other components in liquid milk. However, variations within and between FTIR instruments, even within the same milk testing laboratory, present a challenge to the accuracy of measurement of particularly minor components in the milk, such as individual fatty acids or proteins. In this study we have used Analysis of variance–Simultaneous Component Analysis (ASCA), to monitor the spectral variation between and within each of four different FOSS FTIR spectrometers over each week in an independent milk testing laboratory over 4 years, between August 2017 and March 2021 (223 weeks). On everyday of each week, spectra of the same pilot milk sample were recorded approximately every hour on each of the four instruments. Overall, variations between instruments had the largest effect on spectral variation over each week, making a significant contribution every week. Within each instrument, day-to-day variations over the week were also significant for all but two of the weeks measured, however it contributed less to the variance overall. At certain times other factors not explained by weekday variation or inter-instrument variation dominated the variance in the spectra. Examination of the scores and loadings of the weekly ASCA analysis allowed identification of changes in the spectral regions affected by drifts in each instrument over time. This was found to particularly affect some of the fatty acid predictions.

## Introduction

The goal of quantitative mid-infrared (MIR) analysis is to reproduce the analytical results achieved with accepted standard reference methods. The quantitative analysis of milk components from MIR spectra is based on the direct proportionality between the intensities of the absorbance bands for each component and their concentrations and the path length through the sample. The accuracy of this measurement requires routine calibration of the spectrometers with pre-analysed milk (with chemical reference tests). Signal variations in the interferometer within an instrument over time, between different instruments and between different types of instruments can alter the shapes, intensities and relative intensities of the vibrational mode bands (Pelletier, 2003) which can affect the prediction accuracy, particularly of minor milk components such as individual fatty acids. The accuracy of a predictive calibration is affected both by instrumental factors (Smith et al., 1993) and by the characteristics of the materials used to calibrate the instruments (Kaylegian et al., 2006). Already in the early use of MIR spectral techniques, inherent issues in the stability of predictions between instruments and over time were shown (Biggs, 1978). However, differences in results obtained from different laboratories can also occur because of differences between the reference methods used, and because of failure to achieve good calibrations (Biggs, 1978). In a report on performance of the older generation of milk analysers, it was found that the main problems affecting calibration and accuracy of predictions were inaccurate reference tests, air incorporation, homogenizer inefficiency, mechanical wear, sample cell and filter system, electronics and mechanical maintenance and operator errors (Young, 1978). The newer milk analysers have been engineered with improved designs to minimize these factors; however, some still persist. The small variations in spectra caused by variations in spectrometer parameters such as light source intensity, detector sensitivity and laser stability, and in laboratory environment such as temperature, vibrations, humidity, are minimized by a procedure called Zero-setting (Foss Electric, Hillerød, Denmark) (Hansen, 2020), and by weekly calibration adjustment for fat, protein, lactose and total solids. Manufacturers of other MIR spectrometers used for routine milk analysis similarly incorporate one or more methods to reduce variations within and between instruments.

Differences between the spectrometers, even from the same manufacturer and model in the same laboratory are minimized by routine calibration adjustments. Weekly adjustments on the calibration models are performed to correct or adjust the prediction models used on the different instruments in the laboratory. These adjustments compensate for any week-to-week changes in path length, temperature and humidity variations, mechanical wear, sample handling and minor changes in detector, source and the mechanical and electronic performance. (Young, 1978). Such changes can result in changes in peak intensity or band shape which would render the prediction results inaccurate.

Standardization of MIR spectrometers is particularly necessary for exchange of MIR spectral databases across laboratories and countries. The standardization procedure corrects for systematic variations in intensity due to random variations in linearity of the detectors, or in the relative intensity across the wavelength range from different instrument manufacturers and models. Within the same instrument, the standardization procedure also corrects for path length changes with time, due to erosion of sample cell windows made of CaF_2_ and due to window contamination in the case of diamond sample cell windows. It also corrects for shifts in frequency (or wavenumber), however these are random and if present, would occur on a very minor scale as this is an effect of laser fluctuation, source variation and detector instability, all of which are usually minor compared to other instrument variations.

For the FOSS MilkoScan™ FT1, FT2, FT120, FT + , MilkoScan™ 7 and FT6000 milk analysers, a patented standardization procedure has been developed for regular use which applies a slope and intercept adjustment to the spectra recorded on an instrument to correct for wavenumber (frequency) shift, changes in intensity and changes in linearity due to instrument variations over time. The procedure involves recording a spectrum of a standardization liquid and comparing the intensities and wavenumber positions at two selected wavelengths with those in a standard spectrum (“Master or Gold equalizer spectrum”) stored on the instrument. Any differences between the spectrum of the standardization liquid and gold equalizer spectrum are corrected for by applying four correction factors: A and B for intensity variation, and α and β for wavenumber shifts (Hansen, 2014).

An alternative and non-instrumental standardization method called Piecewise Direct Standardization (PDS) (Wang et al., 1991) was recently used to standardize spectra of samples measured with different makes of instruments (Delta, Bentley, and FOSS) inside a European dairy network. (Grelet et al., 2015a; Grelet et al., 2015b). This standardization aimed to allow spectra from different sources to be pooled and matched to physiological data in a common database to create calibrations predicting cow fertility, health and environmental and feeding indicators. The application of PDS on spectra recorded on 21 different instruments in ten laboratories was found to significantly reduce the RMSE (Grelet et al., 2015b). However, this procedure requires a large amount of post-processing of the spectra and provides retrospective rather than time-based monitoring of instrument performance.

In this paper we describe an innovative approach that covers a different aspect of instrument standardization, namely the routine monitoring of faults or discrepancies in the MIR spectrometers in a single milk testing laboratory over time. This would have a complementary function to the calibration transfer and instrument standardization approaches by monitoring with time the instrument performance. The method relies on measuring spectral variations over time of a pilot sample of milk that is recorded on all the instruments in the laboratory at the same time. A spectrum of the same pilot milk sample is recorded approximately every hour on all instruments in the same laboratory over a period of a week; this is repeated every week using a fresh pilot sample. The effects of day-to-day variation within the individual spectrometers over the week and the variations between the individual laboratory spectrometers, are measured using ASCA (Analysis of variance - simultaneous component analysis) of the spectra (Jansen et al., 2005; Smilde et al., 2005). ASCA is a method used to determine which factors within a fixed effects experimental design are significant relative to the residual error and permits an ANOVA-like analysis even when there are many more variables than samples, as in the case of spectra (Smilde et al., 2005). In this study, the contributors to variation in the spectral intensities over the week were assumed to be instrument and weekday as the main factors, and the interaction between them. The purpose of the study was to explore whether ASCA could provide a useful tool for monitoring and comparing the performance of four MIR milk analysers in a single milk test laboratory in New Zealand. The ability of ASCA to measure changes or differences in the actual spectral output from each instrument allows identification of the source of variation on a weekly basis and thus enable timeous and appropriate intervention.

## Materials and Methods

### Pilot Milk Samples

The pilot milk sample was prepared by combining randomly selected milk samples from three or more different farms from different regions. Every week a new pilot sample was made up as a fresh sample. The aim of sourcing milk samples from different suppliers was to provide the most representative pilot sample, by averaging out milk compositions from different regions of NZ. The pilot sample was then preserved with bronopol and stored in a refrigerator to be used as the single pilot sample for all instruments over that week. For each subsequent week a new pilot sample was prepared. Approximately every hour, a sample of milk from the same pilot sample was introduced to each of the instruments in use, in between measurement of the routine milk samples. This occurred on each MIR analyzer in the laboratory over a period of 1 week from aliquots of the same pilot milk sample. Every week, a fresh pilot sample was prepared for the following week’s measurements.

### MIR Spectra

The MIR spectra of aliquots of the same pilot sample were recorded on between two and four FOSS milk spectrometers at any one time, named in this study as MS1, MS2 (FT6000 models with diamond sample cuvette windows), MS3, MS4 (FT + models with CaF_2_ sampling windows), and MS7 and MS8 (MilkoScanTM 7 with CaF_2_ sampling windows). Although MS1 and MS2 are the same FT6000 model type, MS3 and MS4 are of the same FT+ model type, and similarly MS7 and MS8 are both MilkoScanTM 7, each are individual instruments and will show differences in variations arising from their optical components. For example, the globar light source intensity, detector sensitivity/noise, homogenizer function or interferometer function. Even slight variations in these components of the optical bench will affect the spectral intensities to different extents. These differences are minimized by regular (approximately 6-weekly) instrument standardization procedures (Hansen, 2014). Variations in the predicted milk components are minimized by weekly calibration adjustments by the milk test laboratory of the slope and bias of fat and protein calibration models for a calibration set.

All spectra were recorded between 929 cm^−1^ and 5,000 cm^−1^ at spectral resolution 16 cm^−1^, and ratioed against a water background. The spectra were transformed by an inverse log from transmittance to absorbance. Although the spectrum is measured over 929 to 5,000 cm^−1^, many of these regions were not usable for measurement of milk components. This is mainly because of the intense absorption by water at specific frequencies, although subtracted out, results in random noise between 3,600–3,000 cm^−1^ and between 1,693–1723 cm^−1^. These regions were exluded from the ASCA analysis. In addition, the region between 1785 cm and 1 to 2,600 cm^−1^ was also excluded weak inteference fringes are visible in the region, arising from internal reflection between the inside windows of the samplng cuvette. This region also includes absorption bands by atmospheric CO_2_. These regions were therefore excluded in order to be able to identify variations as being due to changes in instrument parameters within or between instruments or due to other factors such as laboratory environment conditions.

### Fat, Protein, C16:0 and C18:0 MIR Measurements in Pilot Milk Samples

In order to assess the influence of spectral variation on the MIR predictions of the components in the pilot milk MIR predictions, fat measurements (ranging between 3.13–6.54 g/100 ml) and true protein (3.17–4.61 g/100 ml) were selected as examples of major milk components. Also, two fatty acids were selected as minor milk components: the more abundant C16:0 (ranging from 0.94 to 2.04 g/ g milk) and C18:0 (ranging from 0.33 to 0.75 g/100 ml).

### Data Analysis

The first step in ASCA is a decomposition of the variation for every variable (wavenumber) through ANOVA (Jansen et al., 2005; Zwanenburg et al., 2011). We set up a data matrix, X, for each week that contains the spectra of each instrument, and a design matrix that defines the instrument and weekday for each spectrum. An ANOVA is performed for every wavenumber in the FTIR spectra of each pilot sample (week) to determine whether the variation in the spectral data matrix is due to a weekday effect (milk changing or instrument varying over the week), instrument effect (difference between instruments), interactions between instrument and weekday, or other reasons such as noise not described by any of these effects (residual variation). So, for every variable (wavenumber) we define a main effect (the mean), factor effects (instrument and weekday), interaction effects (between instrument and weekday) and a noise or residual term. This results in the definition of different effect matrices:

$$X=X_{mean}+X_{w}+X_{i}+X_{wi}+X_{res}$$ (1)

where w = weekday, i = instrument, wi = interaction between instrument and weekday and res = residuals.

These matrices are made of identical copies of the mean profiles calculated by averaging all the replicates at the different levels of each factor or interaction. For instance, if a factor has two levels, half of the rows of the corresponding effect matrix will contain identical copies of the mean profile of the experiments in which the factor was at level 1; the other half will be made of the average of the remaining signals (i.e., those corresponding to level 2).

Once this decomposition has been done, the effect of the individual design terms is calculated as the sum of squares ( $SSQ_{j}$ ) of the corresponding effect matrix, $X_{j}$ :

$$SSQ_{j}=X_{j}^{2}\;j=i,w,wi$$ (2)

Accordingly, the portion of the total variance in  X , after centering, accounted for by any of the design terms can be calculated by dividing $SSQ_{j}$ by the sum of squares of the mean-centered data ( $X-X_{mean}$ ). The contributions of a factor in the ASCA model can be summarized by dividing the sum of squares of a factor effect matrix by the sum of squares of the mean-centered data.

If a factor/interaction is found to have a significant effect (e.g., by means of permutation tests), PCA is then performed on the corresponding matrix $X_{j}$ to correlate the effect to the variations observed in the spectroscopic profiles.

$$X_{j}=T_{j}P_{j}^{T}+E_{j}\;\;\;\;\;\;\;\;\;j=i,w,wi$$ (3)

where $T_{j}$ , $P_{j}$ and $E_{j}\;$ are the matrices of PCA scores, loadings and residuals, respectively, while the superscript ^T^ indicates matrix transposition. Additionally, to carry out multiple comparisons, when the number of levels for a factor is higher than two or, in general, to graphically visualize the significance of the effect of a design term, it is customary to calculate a new set of scores $T_{j+res}\;$ by projecting the residual matrix onto the PC subspace of the factor/interaction of interest:

$$T_{j+res}=\left(X_{j}+X_{res}\right)P_{j}\;\;\;\;\;\;\;j=i,w,wi$$ (4)

For all the models, to evaluate the statistical significance of the effects, the calculated values of the sum of squares of the corresponding effect matrices were compared to their null distributions, non-parametrically estimated by means of permutation tests (Zwanenburg et al., 2011). Permutation tests were run on the spectra from each week to evaluate the significance of the effect of the different instruments and days in the week, and of their interaction, with 1,000 randomization per model; effects with *p* < 0.05 were deemed significant. The PCA scores and loadings of the corresponding effect matrices were used to highlight differences in the spectra, or changes over time, as influenced by instrument differences or weekday. Box plots were used to monitor differences in values predicted by the calibration models with time.

For each week’s worth of data, outlier removal was performed prior to ASCA calculation, as there were often a small number of spectra with highly anomalous behavior. After calculating PCA models, outliers were identified based on the values of Hotelling T^2^ and Q residuals using the R package “mdatools” (Kucheryavskiy, 2020; Kucheryavskiy, 2021). A square cutoff option was used in which samples with T^2^ > T^2^_lim_ or Q > Q_lim_ (Pomerantsev, 2008), with T^2^_lim_ were calculated using the Hotelling T^2^-distribution and Q_lim_ being calculated at a 99% confidence level based on the corresponding null distributions. These thresholds were chosen so as to include enough spectra to enable comparison of the number of outliers from each instrument, while excluding extreme values to avoid unduly influencing the results of ASCA modeling and sum of squares of the effects.

All computations were performed using the R programming language version 4.0.5 (R Foundation for Statistical Computing, Vienna) and the RStudio integrated development environment (RStudio Team, Boston). The “MetStaT” package was used for ASCA calculations and the package “ggplot2” was used for generating figures.

## Results

### ASCA

A total of 223 weeks’ worth of data comprised the full dataset which spanned from December 2016 to March 2021. Particularly in the winter season (June-August) when fewer milk samples were analysed, and in other periods when one or more instruments were under maintenance, there were not enough instruments active or not enough days in the week with sufficient measurements to perform ASCA. These were excluded from the analysis so that ASCA was performed on the remaining 177 of the 223 weeks.

**FIGURE 1:**
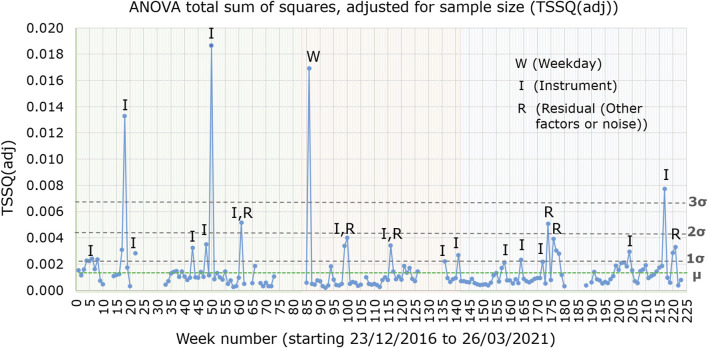
Total sum of squares adjusted for sample size (TSSQadj), for instruments MS1, MS2, MS3, and MS4 from weeks 1–83 (green shaded region), for instruments MS3, MS4, and MS7 over weeks 84–142 (orange shaded region) and for instruments MS3, MS4, MS7, and MS8 over weeks 143–223 (blue shaded region). Weeks in which the TSSQ (adj) exceeded thresholds of one, two and three times the SD (σ) of the 177 weeks TSSQ (adj) values are labelled with the main contributing effects. The mean TSSQ (adj), labelled as µ, is represented by a green horizontal dotted line.

The total sum of squares (TSSQ) for each of the 177 weeks, obtained from the ANOVA calculation of the ASCA algorithm are plotted in Figure 1 over the time period December 2016 to March 2021. The TSSQ was adjusted for sample size to TSSQ (adj), as the number of samples over the measurement period varied each week between 202 and 1,324, depending on time of year (fewer samples in winter season) or whether instruments were undergoing maintenance. The plot of TSSQ (adj) in Figure 1 shows the overall variance for every week for all the instruments in the laboratory and serves as a useful monitor of instrument performance and/or laboratory stability.

The shaded regions in the plot indicate changes in which laboratory instruments were used. Between weeks 1 and 83, the four instruments MS1, MS2, MS3, and MS4 were active, while over weeks 84–142 only instruments MS3, MS4 and MS7 were active. From weeks 143–223, the four instruments MS3, MS4, MS7, and MS8 were active. The dashed lines indicate the thresholds for one (0.0022), two (0.0044) and three (0.0066) standard deviations of all 177 weeks’ TSSQ (adj) values. These thresholds can be selected to flag when the overall spectral variance deviates from the norm. The mean TSSQ (adj) (0.0015) is also indicated on Figure 1 as a green dotted line.

For 130 of the 177 weeks (74%) the TSSQ (adj) was below the mean. In 28 of the 177 weeks (15%) the TSSQ (adj) was above one SD (σ) of the 177 weeks’ TSSQ (adj) values. Of these 28 weeks, seven exceeded two SD’s (4% of the 177 weeks) and six exceeded three SD’s (3% of the 177 weeks), with 15 exceeding only one SD (8% of the 177 weeks). The weeks with TSSQ (adj) exceeding one, two or three SD’s are labelled according to the major contribution from one or more of weekday effect (W), instrument effect (I), or residual effect (R). There was no correlation between the TSSQ (adj) exceeding one, two or three times the SD of the 177-weeks TSSQ (adj) values with time of year or with season.

**FIGURE 2:**
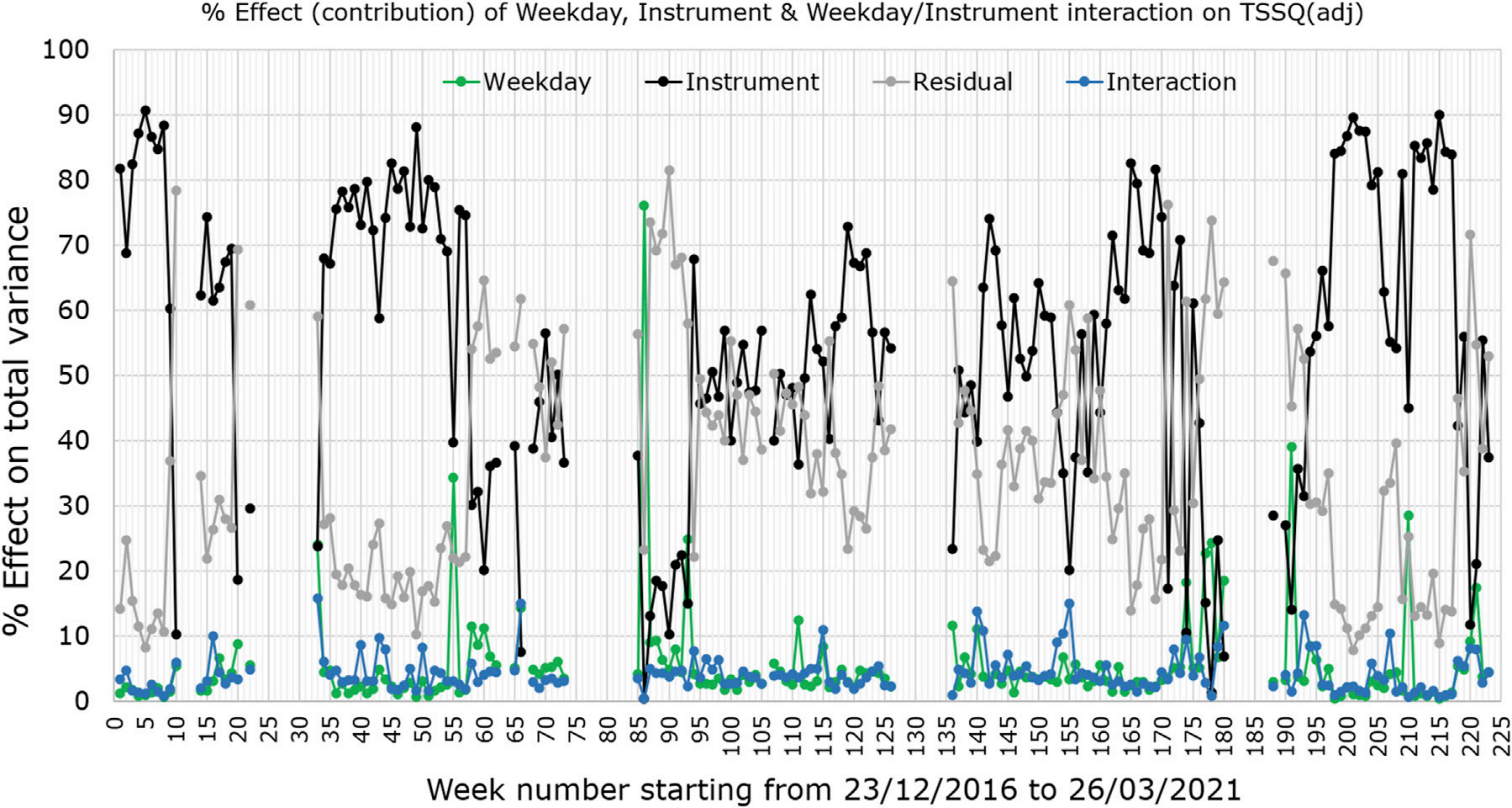
Weekly percentage contribution of instruments (black trace), weekdays (green trace) and weekday/instrument interactions (blue trace), to the total variance (TSSQ, adjusted for sample numbers) in the pilot sample spectra. Also plotted is the residual effect (grey).

Figure 2 shows the percentage contribution to the total SSQ (representing the total variance) for each week, by the instrument effect (black trace), weekday effect (green trace), weekday/instrument interaction (blue trace) and residual factors/noise (grey trace). Evident from the graph is that weekday changes originating from the sample itself or within each instrument, and weekday/instrument interactive effects contribute very little to the overall variance. Differences between instruments and residual variations form the major contributions; only three of the 177 weeks showed greater contribution from weekday variation than instrument effects. 53 of the weeks have residual effects contributing more than either instrument or weekday effects to the TSSQ (adj).

In all 177 weeks measured with ASCA the contribution of the instrument effects to the TSSQ (adj) was significant, having *p* < 0.05. The weekday effects were also significant except for two of the weeks: with only weeks 137 and 188 having *p* > 0.05. Interaction effects between weekday and instrument were significant for all weeks except for 43 and 177. Those weeks exceeding one, two and three standard deviations of the 177 weeks’ TSSQ (adj) values are listed in Table 1, with the number of SD’s indicated in the fifth column from the right. Also listed are the total number of samples (N) for each week and the ASCA output of percentage contribution from each of the effects: weekday (SSQ day), instrument (SSQ instr), weekday/instrument interaction (SSQ inter), and the residual variance due to other factors and noise (SSQ resid).

**TABLE 1:** List of weeks in which the TSSQ adjusted for sample number [TSSQ (adj)] exceeded one, two or three times the standard deviation of the 177 weeks, given in the column fifth before the last. N is the total number of samples in each week. The percentage that each of the factors contribute to each weekly total SSQ [TSSQ (adj)] are given as SSQ (day), SSQ (instr) and SSQ (inter) (weekday/instrument interaction). The residual variance due to other factors is SSQ (resid). The number of standard deviations (SDs) by which the SD’s in each of MIR predicted values of fat, protein, C16:0 and C18:0 exceed the SD’s of the 177 weeks by 1, 2, or 3 SD’s are given on the four rightmost columns of the table.

Week No	No. samp	SSQ (day)	SSQ (instr)	SSQ (inter)	SSQ (resid)	TSSQ	TSSQ (adj)	No of SDs	SDs^a^ (fat)	SDs^a^ (prot)	SDs^a^ (C16:0)	SDs^a^ (C18:0)
4	853	0.8	87.2	1.35	11.46	1.92	0.0023	1	1	3	3	3
5	712	0.88	90.65	1.17	8.17	1.57	0.0022	1	−	1	2	3
6	765	1.39	86.65	2.53	11.1	1.82	0.0024	1	−	1	3	3
8	907	0.71	88.44	0.82	10.61	2.12	0.0023	1	1	1	2	3
17	578	6.67	63.58	4.5	30.9	1.69	0.0029	1	1	1	2	3
18	725	3.36	67.46	2.7	28.01	9.48	0.0131	3	−	3	3	3
22	252	5.46	29.55	4.81	60.75	0.71	0.0028	1	3	1	3	3
43	1125	4.89	58.81	9.67	27.24	3.63	0.0032	1	−	2	2	3
48	1164	2.87	72.83	4.93	19.81	4.06	0.0035	1	−	2	3	3
50	1306	3.03	72.61	8.24	16.91	24.18	0.0185	3	−	3	3	3
61	469	6.86	36.08	4.57	52.6	2.42	0.0052	2	1	3	1	2
86	514	76.04	0.37	0.39	23.3	8.69	0.0169	3	3	1	3	3
99	1122	1.72	56.96	2.63	40.03	3.75	0.0033	1	1	1	1	3
100	1002	3.41	40.05	2.7	55.25	3.95	0.0040	1	1	1	2	3
116	848	2.21	40.25	2.96	55.26	2.87	0.0034	1	3	1	3	3
141	671	3.76	63.54	10.86	23.3	1.77	0.0026	1	−	1	1	3
164	747	2.24	35.11	4.03	58.76	2.22	0.0023	1	1	2	2	3
174	512	18.28	10.49	9.51	61.35	2.6	0.0051	2	−	1	2	3
176	504	5.16	42.66	6.79	49.39	1.94	0.0039	1	−	1	2	3
177	372	22.71	15.09	2.85	61.74	1.11	0.0030	1	−	−	2	3
178	334	24.32	1.35	0.75	73.85	0.94	0.0028	1	−	1	1	3
204	1144	3.12	79.21	5.77	13.06	3.34	0.0029	1	−	1	3	3
217	793	1.32	83.94	1.07	13.74	6.13	0.0077	3	−	2	3	2
220	749	9.16	11.72	8.14	71.66	2.13	0.0028	1	−	1	2	3
221	578	17.37	21.05	7.97	54.69	1.89	0.0033	1	−	1	2	3

a Number of standard deviations exceeding the average SD of 177 weeks, by the MIR-predicted values of fat, protein, C16:0 and C18:0 in each of the 25 weeks having TSSQ (adj) > 1 SD of the 177 weeks.

Of interest is how the TSSQ (adj) affects the accuracy of the milk component MIR predictions of fat, true protein, C16:0 and C18:0. The columns on the right-hand side of Table 1 give the number of SD’s by which each of the MIR predicted values of fat, protein, C16:0 and C18:0 exceed the average 177-weeks SD of each component: i.e., by one, two or three SD’s. Of the 25 weeks shown in Table 1 having TSSQ (adj)>1SD, the number of weeks in which the SDs of the fat predictions overall exceed one or more SDs is 10. For true protein, 24 of the 25 weeks have predicted values with SD’s exceeding the average SD by one or more, while for C16:0 and C18:0 this occurs in all 25 weeks. This is not unexpected, as predictions of more minor components would be expected to be more sensitive to variations between instruments or within each instrument through the week, whether this is due to changes in the pilot sample, other effects such as laboratory environment or instrument variations.

### PCA of Weeks Exceeding Three Standard Deviations

**FIGURE 3:**
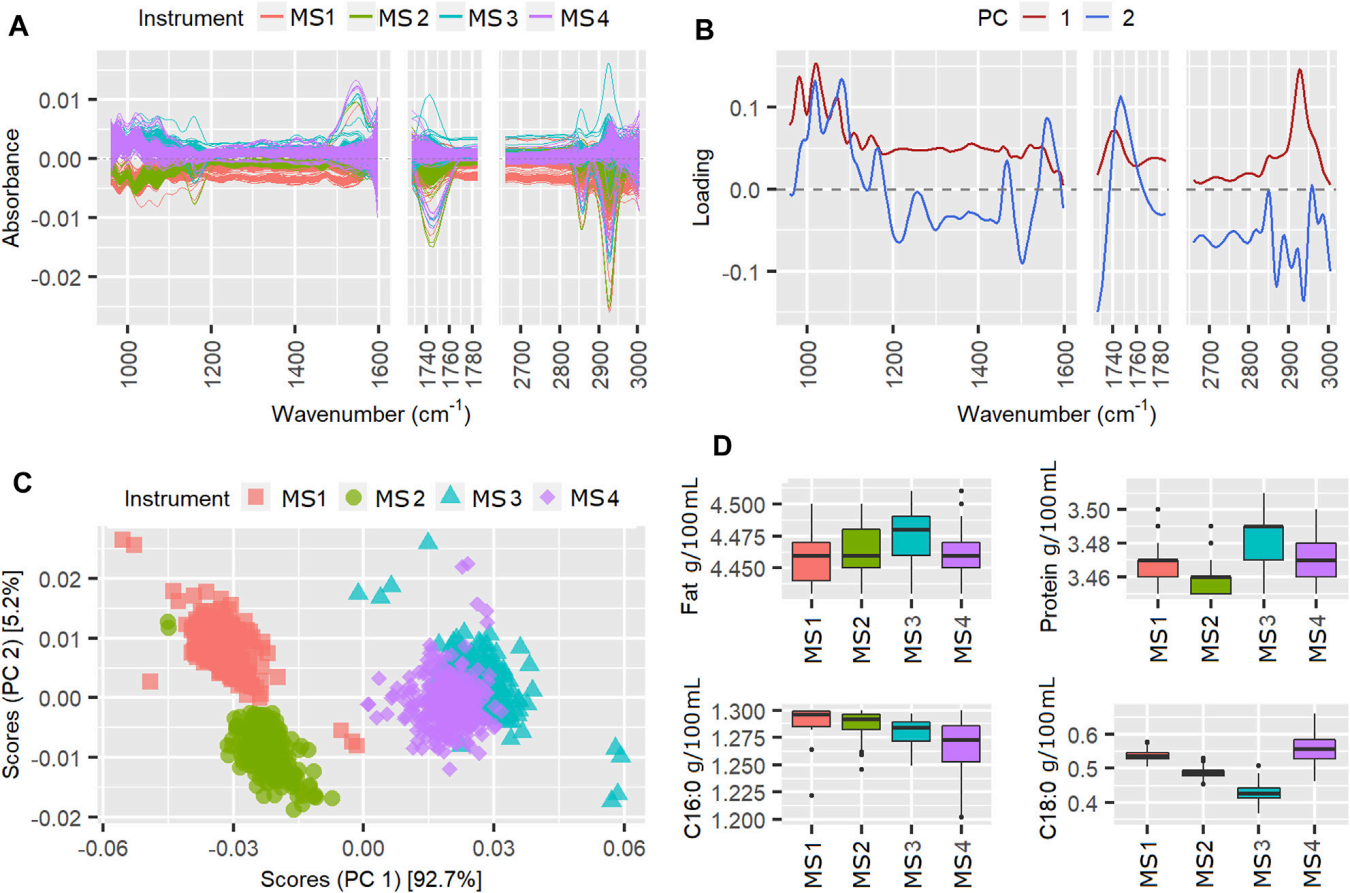
Mean centered spectra **(A)**, instrument loading plots **(B)** and scores **(C)** for pilot spectra from week 50, in which the TSSQ (adj) exceeded 3 standard deviations. Boxplots **(D)** show the predicted fat, protein, C16:0 and C18:0 values from each instrument and their mean values and standard deviations (SDs), marked * for 1SD, ** for 2SDs and *** for 3SDs greater than the SD of the 177 weeks TSSQadj values.

**FIGURE 4:**
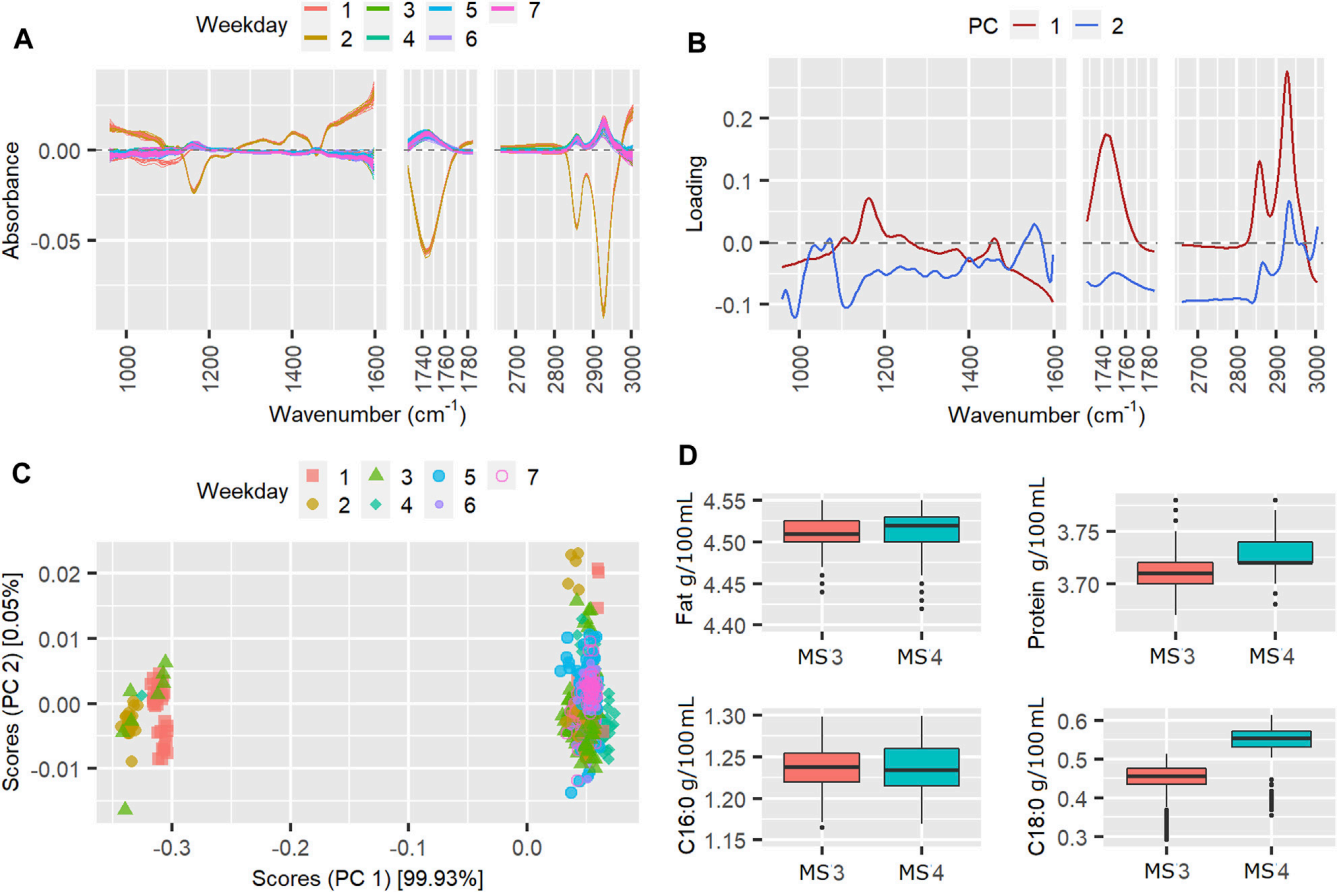
Mean cenered spectra **(A)**, instrument loading plots **(B)** and scores **(C)** for pilot spectra from week 86, in which the TSSQ (adj) exceeded 3 standard deviations. Boxplots **(D)** show the predicted fat, protein, C16:0 and C18:0 values from each instrument and their mean values and standard deviations (SDs), marked * for 1SD, ** for 2SDs and *** for 3SDs greater than the SD of the 177 weeks TSSQ (adj) values.

**FIGURE 5:**
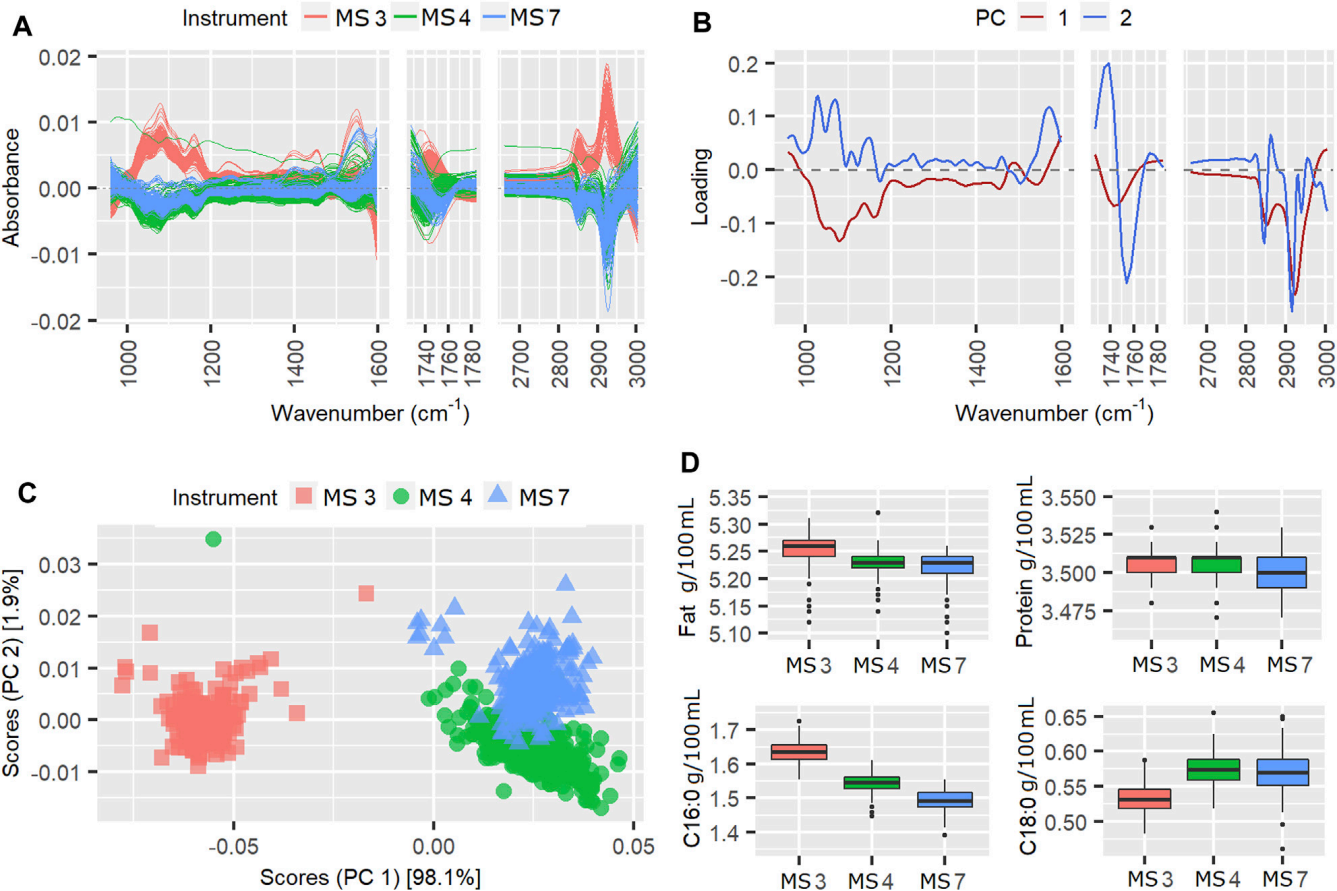
Mean centered spectra **(A)**, instrument loading plots **(B)** and scores **(C)** for pilot spectra from week 217, in which the TSSQ (adj) exceeded 3 standard deviations. Boxplots **(D)** show the predicted fat, protein, C16:0 and C18:0 values from each instrument and their mean values and standard deviations (SDs), marked * for 1SD, ** for 2SDs and *** for 3SDs greater than the SD of the 177 weeks TSSQ (adj values.

The instrument scores and loadings plots from the ASCA can provide more information about the spectral variations over the weeks with TSSQ (adj) exceeding one or more standard deviations (SD). Of particular interest are the weeks showing TSSQ (adj) exceeding the three SD threshold. The scores and loadings of three of these weeks with such TSSQ (adj): weeks 50, 86, and 217, are shown as examples in Figure 3 (week 50), Figure 4 (week 86), and Figure 5 (week 217). Also plotted are the spectra after mean centering and boxplots for the MIR-predicted components fat, true protein, C16:0 and C18:0. The mean and standard deviations are indicated in the boxplots for each component for that week, with a number of asterisks that indicate the number of SDs by which each predicted component exceeds the SD of the 177 weeks TSSQ (adj) values (* for 1 SD, ** for 2 SDs and *** for 3SDs). Breaks in the plots of the spectra and loadings show the spectral regions excluded from the ASCA analysis. The scales of the intensity axis of the box plots have been expanded over reduced regions to exclude extreme outliers, in order to more clearly compare the medians inter quartile ranges (IQR) and whiskers.

According to Table 1, the instrument effect in week 50 contributed 72.6% of the TSSQ (adj). The mean centered spectra in Figure 3A show clear differences in the spectral intensities, particularly between the MS3, MS4 instruments and MS1, MS2 instruments, and particularly in the spectral region 930–1,200 cm^−1^. The PCA scores in Figure 3C show clear separation along PC1 for the two sets of instruments. This can be explained by differences in FOSS instrument models; MS3 and four are FT6000 models with CaF_2_ windows while MS1 and MS2 are FT + models with diamond windows. Additionally, along PC2, MS1 and MS2 are more separated than MS3 and MS4. Ideally all four scores should overlap as the six-weekly standardization procedure adjusts the slave spectra of each instrument to match a master spectrum. The PC1 loading in Figure 3B shows that the main difference between the two spectrometer models are overall intensity, possibly due to pathlength differences, with the CaF_2_ windows in MS3 and MS4 possibly eroded at this point to a slightly wider pathlength. The regular wavelike features in the loadings may be due to interference patterns from internal reflectance in the cell windows. These interference patterns were also seen in the spectral regions between 1730 and 2,650 cm^−1^ which were excluded from the analysis for this reason (besides this region displaying atmospheric CO_2_ bands).

The boxplots of the MIR predicted components in Figure 3D, however, do not correspond with the PCA observations of higher fat for MS3 and MS4 compared with MS1 and MS2. This may be because the weekly calibration adjustments for fat have compensated for the spectral differences. The instrument differences for week 50 do, however, result in the true protein, C16:0 and C18:0 values for this week showing SDs three times higher than the 177-weeks average SD, while the fat predictions were not affected.

The results for week 86 are given in Figure 4. In week 86, the weekday effect contributed 76% to the TSSQ (adj). Being the winter season, only two spectrometers, MS3 and MS4 were active in this week.

The mean-centered spectra clearly show a subset of spectra from three of the weekdays that markedly differ from the others. This is also seen in the weekday scores plot. PC loading 1 is mostly represented by fat bands (C-H stretching of lipids 2,550–2,962 cm^−1^, C=O stretching of fatty acids at 1745 cm^−1^ and C-O-C stretching of fatty acid esters at 1,160 cm^−1^) (Grelet et al., 2015b). The separation of the scores according to these differentiates some of spectra in weekdays 1, 2, and three from the rest of the spectra in days 1, 2, and 3, and all the spectra in days 4–7. The weekday effect accounted for 76% of the variance compared to around 0.4% from instrument effects and weekday/instrument interactions. This implies that all both instruments, MS3 and MS4 underwent changes in weekdays one to three that resulted in a bigger effect than any differences between the instruments. These weekday differences result in the MIR-predictions of fat, C16:0 and C18:0 having SD’s more than three times the SD of the 177-weeks TSSQ (adj). The protein was less affected, exceeding only one SD this week; this is also evident in the loadings which represent mainly fat and fatty acids.

The results for week 217 are shown in Figure 5. During week 217 three instruments were active and the instrument variation contributed 83.9% to the TSSQ (adj). The separation of instrument scores in the scores plot in Figure 5C shows that the spectra of instrument MS3 are consistently different from those of the MS4 and MS7 instruments. All three instruments are the same model, however, the higher negative PC1 spectral loadings appear to show that MS3 has generally higher spectral intensities than the other two (Figure 5A). This difference translates into higher predicted values for fat and C16:0 as shown in the boxplots (Figure 5D), but the MS3 C18:0 values are lower. The differences in the spectra have likely been compensated for by the weekly fat and protein calibration adjustments, given that the SDs for fat and protein are below one SD of the 177-weeks average. The MS3 spectral differences do affect the variation in measurement for true protein, however, and also affect the C16:0 and C18:0 measurements, with the SD at three times and twice the 177-weeks average, respectively.

### PCA of a Series of Four Successive Weeks: 193 to 196

**FIGURE 6:**
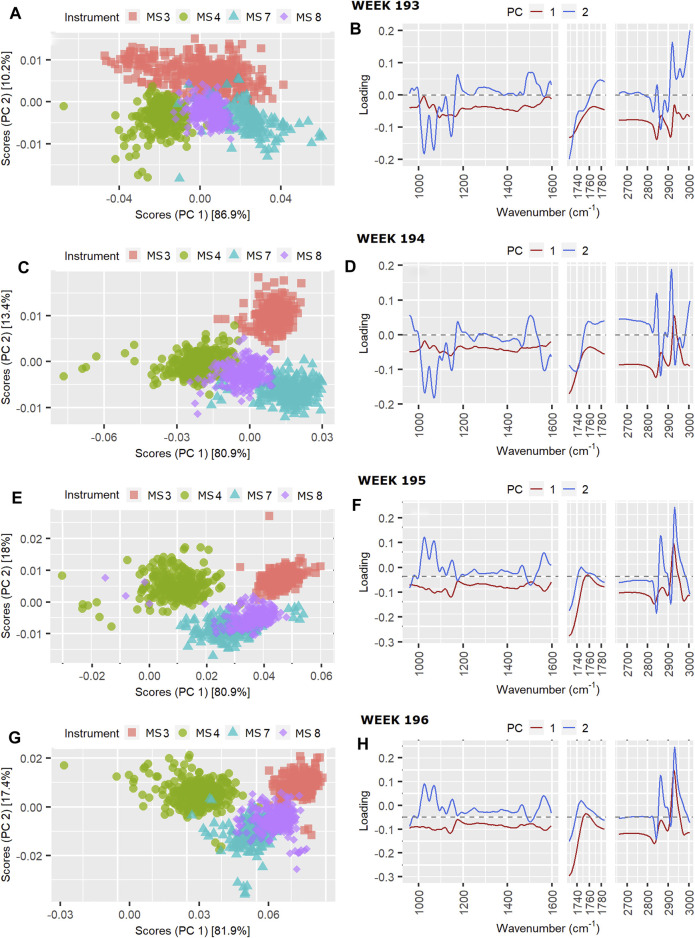
Instrument scores and loading plots, respectively, for pilot spectra from four successive weeks: **(A)** and **(B)** week 193, **(C)** and **(D)** week 194, **(E)** and **(F)** week 195 and **(G)** and **(H)** week 196.

**FIGURE 7:**
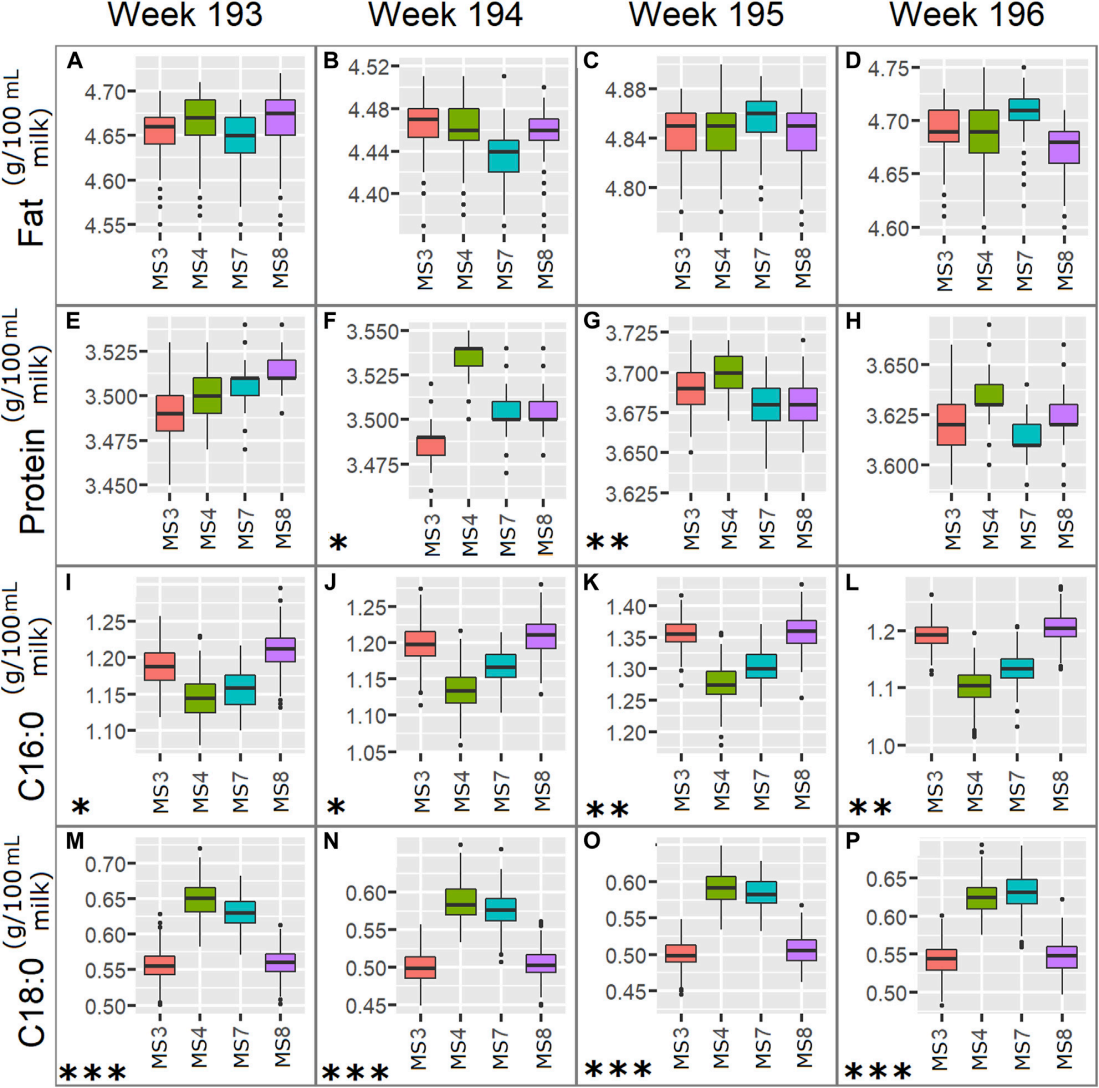
Boxplots for fat, true protein, C16:0 and C18:0 predicted from pilot spectra using instruments MS3, MS4, MS7, and MS8, over successive weeks that had low TSSQ: week 193: **(A)** Fat, **(E)** True protein, **(I)** C16:0, **(M)** C18:0; week 194: **(B)** Fat, **(F)** True protein, **(J)** C16:0, **(N)** C18:0; week 195: **(C)** Fat, **(G)** True protein, **(K)** C16:0, **(O)** C18:0 and week 196: **(D)** Fat, **(H)** True Protein, **(L)** C16:0, **(P)** C18:0. The plots for each week show the median, IQR and variability outside the upper and lower quartiles for each component. Asterisks represent the number of SD’s, marked * for 1SD, ** for 2SDs and *** for 3SDs, by which the predicted component exceeds the average of all 177-weeks average SD.

Of interest for routine monitoring of instrument performance are changes in contribution from instrument effect on TSSQ (adj) over successive weeks. An example of such a change is seen in the sharp increase in instrument effect (black trace) on the TSSQ (adj) in Figure 2 between weeks 193 and 196. These weeks were selected as an example because over this period, the same four instruments were in use and the TSSQ (adj) was well below the mean. A PCA could show useful insight into the observed increase in instrument effect, while the corresponding boxplots would show how this affects the MIR-predicted component values. The PCA scores and loadings are plotted for weeks 193–196 in Figure 6, and the corresponding boxplots for the four MIR-predicted components are given in Figure 7.

The scores can be seen to drift slightly over the first 3 weeks, with the biggest change between instrument scores and loadings occurring between weeks 194 and 195, while those for weeks 195 and 196 are similar. Differences in scores between week 193 and 194 are mainly along PC1 and mainly for MS3 (Figures 6A,C), with the main difference in the PC1 loadings between weeks 193 and 194 being in the relative intensities of the lipid C-H stretch modes at 2,856 and 2,926 cm^−1^ (Figures 6B,D), and the protein amide II band intensity around 1,550 cm^−1^. This is consistent with an increase in the instrument effect from 31.5% in week 193 to 53.7% in week 194, and an increase the weekday effect from 3.1% in week 193 to 8.5% in week 194. The difference observed in the loadings affect the protein prediction by MS3 (Figure 7B), however, it does not result in a SD above the 177-weeks average.

Between weeks 194 and 195 there is a noticable drift in the scores for MS4 away from those of the other instruments, and further up PC2 (Figures 6E,G). The loadings show that these differences are due to clear changes in relative intensities between the C-H stretching region at higher wavenumbers 2800–3,000 cm^−1^ typical for fat and the C-O stretching and C-H deformations at lower wavenumbers between 1,000–1,100 cm^−1^, representing mostly lactose (Figures 6F,H). These changes result in a slight increase in fat prediction for MS3 relative to the other instruments (Figures 7K,L), and an increase in SD from one to two SD’s above the 177-weeks mean in predicted values for C16:0, indicated by single asterisks in the boxplots in Figures 7K,L for weeks 195 and 196. These changes also correspond with a small increase in instrument effect, from 53.6% in week 194 to 56.1% in week 195 (Figure 2). At the same time the weekday effects decrease from 8.5% in week 194 to 6.3% in week 195, corresponding with reduced spread of scores for MS3 along PC1 (Figures 6C,E). The changes in scores plots and loadings from weeks 195–196 are small, however, the contribution of instrument effects increases from 56.1 to 66.1% between weeks 195 and 196, while the weekday effects decrease from 6.3 to 2.3%. The change in relative contribution of these effects can be seen in a small decrease in spread over PC1 of the scores in Figure 6G for week 196.

## Discussion

Plotting the TSSQ (adj) for each week with time (Figure 1) presents an overview of the overall variance of the active instruments in the laboratory over the time period December 2016 to March 2021. This 4 year time record enables a robust measure of the SD expected over all seasons, and can be used to monitor instrument performance and/or laboratory environment stability with time. When the TSSQ (adj) is flagged as exceeding one, two or three times the SD of the 177 week period, the contribution of instrument, weekday, interaction between these or residual effects can be examined to identify the source of variation. The plot showing the contribution from these effects in Figure 2 shows that over the 4 years, the major contributions to the TSSQ (adj) of each week are differences between instruments and residual effects.

Factors contributing to residual effects include ground vibrations, electronic gain settings, cell temperature, instrument temperatures and operator changes (Young, 1978). Controlling the lab temperature and humidity aims to minimize variations in these. Instrument effects have multiple sources of variations. The detectors used in the MIR milk instruments are DTGS (deuterium triglyceride sulphate) thermal detectors that convert thermal energy to electrical signal; they respond to temperature by changing their capacitance which is measured as a voltage change. Again, controlling the lab temperature and humidity minimizes variations in these detectors, however, noise from the IR source due to random photons and thermodynamic noise in interaction with these photons can also affect both the intensity of, and noise in the signal (King et al., 2004) and so contribute to residual variation. In addition, pathlength changes are commonly caused by build-up of protein and foreign material on the windows of diamond sample cells, or gradual erosion of sample cell windows made of CaF_2_. This results in changes in path length, which also greatly affect the IR signal/noise ratio in aqueous systems, (Jensen and Bak, 2002), and can cause a calibration shift.

The homogenizers on each individual instrument wear at different rates, depending on the number of samples through each instrument. The effects of variations in these instrument components on the spectra are minimized by routine standardization procedures, typically every 6 weeks, and by monitoring a homogenizer index which measures the efficiency as an approximate prediction of one of the fat globule distribution parameters (FOSS, private communication).

Considering these possible sources of variation, instrument differences would therefore be expected to have the greatest effect on variations in the pilot milk spectra over the week, with residual effects also contributing to a large extent. The *p*-values>0.05 confirming significance of the instrument effects throughout all 177 weeks confirms this, while the weekday variations within each instrument were significant for only two of the 177 weeks. Recent work on minor milk components such as milk urea has also shown the impact of inter instrument differences on IR predicted results (Wood et al., 2020; Portenoy et al., 2021).

Of the 25 weeks in Table 1 with TSSQ (adj) exceeding one or more of the 177-weeks average SD, only two of the 177 weeks (1% of the time) had TSSQ (adj) values above 2SD’s and in only four (2%) of the time) the TSSQ (adj) exceeded 3SD’s (Table 1). The TSSQ (adj) in the other 19 weeks the exceeded only one SD above the 177-weeks mean (11% of the time). This is a relatively low rate which shows that the weekly calibration adjustments and regular instrument standardization procedures are effective in adjusting for instrument drift and maintaining the inter-instrument and intra-instrument variances below one SD of the 177-weeks mean, during 152 of the 177 weeks (86% of the time). Of the 25 weeks in Table 1, the instrument effect dominated the TSSQ (adj) in 13 weeks (52%), while the weekday effect dominated once (4% of the time) while residual effects dominated in 11 weeks (44% of the time).

The 25 week period in which the TSSQ (adj) > 1SD of the 177-weeks average was found to affect the mean and SD of the four components fat, protein, C16:0 and C18:0 to different extents. In this period, the predicted fat SD exceeded the 177-weeks average by one or more in only 10 of the 25 weeks, while for the true protein this occurred in 24 of the 25 weeks. The prediction of the less abundant fatty acids, C16:0 and C18:0, was affected in all 25 weeks, with C18:0, the least abundant consistently showing SD’s three times higher than the 177-weeks average. Predictions of other fatty acids, not discussed here, were also found to show differing extents of SD’s over this period, and greater than those shown by the major milk components fat, protein, lactose and total solids. We thus note the relevance of this more sensitive monitoring approach considering the recent trend towards deployment of predictive models focused on greater use of IR data of milk (Grelet et al., 2017). Recent work on minor milk components such as milk urea has also shown the impact of inter instrument differences on IR predicted results. (Wood et al., 2020; Portenoy et al., 2021).

The PCA scores and loadings obtained from the ASCA analysis of the spectra are useful for monitoring instrument drift with time. This was shown in the example of four successive weeks 193–196, during which a marked increase in instrument effect from 31 to 66% was observed, while at the same time the residual variation contribution decreased from 53 to 29%, while weekday or within-week variations showed no trend, instead signaling spread of weekday scores along PC1. The scores and loadings can be monitored to signal drifts beginning to occur in individual instruments week by week. Weekly calibration adjustments of the instruments allow adjustment of bias and slope of the fat, protein, total solids and lactose calibrations in the laboratory and thus compensate for differences in all the milk component predictions that may arise through weekly changes in instrument performance. The ASCA scores are especially sensitive to differences in spectral intensities of the different instruments on different weekdays, and to changes with time in relative intensities over the spectral region. Monitoring the ASCA scores and loading plots could provide a useful indicator of the extent of instrument drift, and signal when standardization of the instruments would be necessary rather than adjusting the calibration to compensate for these changes. We suggest such an approach could be used in conjunction with recent advances in instrument standardization that have allowed calibrations to be deployed across networks of instruments from different manufacturers (Grelet et al., 2021).

Monitoring the boxplots of predicted components could be useful for testing the effectiveness of the calibration adjustments of the major components and whether these improve the predictions of less abundant components such as individual fatty acids or indirectly-measured traits. Comparison of the boxplots with the score plots and loadings are also useful for evaluating when calibration adjustments are compensating for instrument differences to an extent that instrument signal standardization is necessary.

## Conclusion

We have described the novel use of ASCA on the spectra of pilot test milk samples over time as a new approach for routine monitoring of instrument performance in a milk testing laboratory. Plotting of the scores and loadings derived from the ASCA effect models, the mean centered spectra and boxplots of the MIR-predicted components provides a useful overview of the weekly performance of the spectrometers in the laboratory, in terms of day-to-day variations in spectral intensities, differences arising between spectrometers and to what extent the spectral variance shows residual effects, not explained by these two effects, such as changes in laboratory environment or unexplained noise. This can be particularly useful to flag unexpected laboratory environment changes or weekly instrument changes that may affect the accuracy of the MIR-predicted milk components. Weekly monitoring of these plots can also serve as an indicator for when instrument standardization of one or more instruments is necessary, and can evaluate when the weekly calibration adjustments may be compensating for instrument differences. Comparison of the boxplots with the score plots and loadings is also useful to signal the effectiveness of instrument standardization and the weekly calibration adjustments, especially with the trend towards greater use of IR data for predicting milk components and other relevant traits present in lower levels.

## Data Availability

The raw data supporting the conclusions of this article will be made available by the authors, without undue reservation.
